# Microfabricated electrochemical aptasensing chip modified with dual-function antifouling linker for single-drop label-free assay of oxytetracycline in milk

**DOI:** 10.1007/s00604-025-07387-4

**Published:** 2025-07-28

**Authors:** Christina Bizinti, Dionysios Soulis, Dimitra Kourti, Georgia Geka, Christos Kokkinos, Michael Thompson, Lidia Nemtsov, Thanassis Speliotis, Anastasios Economou

**Affiliations:** 1 Department of Chemistry, National and Kapodistrian University of Athens, Athens, 15771 Greece; 2 Department of Chemistry, University of Toronto, 80 St. George St, Toronto, ON M5S 3H6 Canada; 3 Institute of Nanoscience and Nanotechnology, N.C.S.R. Demokritos, P.O. Box 60037, Agia Paraskevi, 15310 Greece

**Keywords:** Oxytetracycline, Aptasensor, Antifouling linker, Electrochemical detection, Differential pulse voltammetry, Microfabrication

## Abstract

The fabrication and characterization of a novel integrated electrochemical aptasensing device and its application to oxytetracycline (*OTC*) determination in milk is described. The microfabricated three-electrode chip is composed of gold working and counter electrodes and a silver reference electrode deposited on a Kapton film by physical vapor deposition. The working electrode is modified with *α-*lipoic acid-NHS, an antifouling linker, onto which an amine-modified *OTC*-specific aptamer is further attached. The label-free assay of *OTC* involves incubation of the sample with the linker/aptamer bioconjugate immobilized on the working electrode and monitoring of the *OTC*-aptamer binding event by means of the electrochemical response of the [Fe(CN)_6_]^3−^/[Fe(CN)_6_]^4−^ redox couple, The decrease of the signal magnitude, induced by blocking the diffusion of the probe, is related to the concentration of *OTC*. The limit of detection for *OTC* is 7 ng mL^−1^ and the inter-sensor reproducibility is 13.7%. The sensor is applied to milk samples with recoveries between 107 and 110%. This aptasensing chip demonstrates strong potential for rapid on-site detection of *OTC* in the food industry due to its high degree of integration, easy functionalization, and potential for single-drop operation.

**Graphical abstract:**

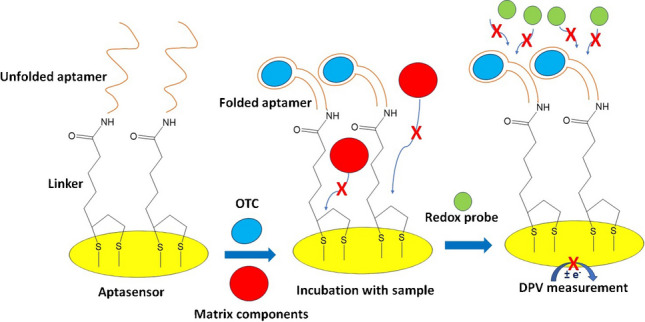

**Supplementary Information:**

The online version contains supplementary material available at 10.1007/s00604-025-07387-4.

## Introduction

Milk is a widely consumed foodstuff worldwide and is particularly important for its high nutritional value and for its key role in the worldwide economy. A major concern about milk is the contamination with antibiotics administered to dairy-producing animals to treat, and prevent from, various bacterial diseases as well as to promote growth of food-producing animals. Overuse or misuse of antibiotics leads to foodstuff contamination affecting the consumers’ health [1, 2]. In particular, the occurrence of veterinary drug residues in animal-derived foods produces a significant health risk for the consumers because of the emergence of microbial resistance hypersensitivity and allergic reactions [2]. Therefore, national and international legislations have been established to closely monitor antibiotic residues in food. The EU European Medicine Agency (EMA) and Codex Alimentarius have set maximum residue limits (*MRLs*) of antibiotics in milk as a precautionary value to ensure consumers’ health with values ranging from 4 to 200 μg/kg [1]. Tetracyclines is a class of antibiotics that comprises several natural or synthetic compounds which are widely used in veterinary practice due to their wide spectrum of activity and low cost [3]. Among them, oxytetracycline (*OTC*) is one of the most widespread antibiotics and, thus, the EU and China have regulated the *OTC* levels in milk at 100 μg/kg while the Food and Drug Administration (FDA) in the USA has set a *MRL* of 400 μg/kg [4].

Among the various approaches developed to detect and quantify antibiotic residues in food, the most prominent are immunoasays (such as enzyme-linked immunosorbent assay (ELISA), radioimmunoassay (RIA), fluorescence immunoassay (FIA), and colloidal gold lateral flow immunochromatographic assays), high performance liquid chromatography (HPLC)-based methods and microbiological methods [1, 5]. However, immunoassays rely on antibodies as biorecognition elements; these molecules are expensive; their activity changes with time and are sensitive to pH and temperature variations [1, 5]. On the other hand, HPLC methods require sample preparation, expensive instrumentation, extensive laboratory facilities, and skilled personnel, while microbiological inhibition tests are slow and lack selectivity [1, 5].

Biosensors constitute an innovative alternative technology to develop assays for antibiotic residues [6, 7]. Biosensors generate a measurable signal from the interaction between a biorecognition element with the target analyte and have many advantages, compared to the aforementioned analytical approaches: they are portable, have low power requirements, can provide real-time analysis, do not require specific skills to operate, and are suitable for on-site analysis. Traditionally, antibiotics have been measured using immunosensors, exploiting antibodies as the biorecognition element due to their availability and selectivity towards a broad range of target molecules [8]. Recently, several aptamer-based biosensors (aptasensors) have been developed as alternative to immunosensors for antibiotic detection [9–11]. Aptamers are single-stranded DNA or RNA oligonucleotides, which can bind with high affinity, selectivity, and sensitivity to a wide range of target molecules, like nucleic acids, proteins, metal ions, and other small molecules. Aptamers have many advantages compared to antibodies since they can be produced at low cost, can be easily modified with labeling moieties, have longer shelf lives and are less prone to loss of activity on labeling and to denaturation in complex matrices [5, 9–11]. Combining aptasensors with electrochemical signal transduction is advantageous because of the portable and low-cost instrumentation needed, the high sensitivity and the scope for device miniaturization [12], therefore many electrochemical aptasensors have been developed for the assay of antibiotic residues [13, 14]. Yet, a major problem in electrochemical biosensing is that two or more electrodes are required for the transduction, therefore the issue of device integration in a single self-contained package is of outmost importance for the development of truly portable and miniaturized devices [15, 16]. Although many biosensors use enzymatic, fluorescent, chemiluminescent, electrochemical and radioactive labels to generate a measurable signal, there are several complications that may arise from the use of such labels; they require careful handling (in case of radioactive and fluorescent reagents), are prone to loss of functionality during the labeling process and complicate the analytical protocol leading to lower precision. Label-free biosensors, despite being generally less sensitive than labeled ones, provide more cost-effective, rapid, and simple analysis [17, 18].

Regarding the detection and quantification of *OTC* in milk, only a few label-free electrochemical aptasensors have been reported (Table S1, Supplementary Material). The principle of all the existing methods is based on (a) the immobilization of an *OTC*-specific aptamer on the surface of a working electrode, (b) the recording of the signal of a diffusion-controlled electrochemical probe, (c) the capture of the target molecule from the sample solution, (d) the recording of the (weaker) signal of the electrochemical probe after the aptamer-target binding, and (e) the calculation of the differential signal resulting from the inhibition caused by the binding event. To this end, Akbarzadeh et al. have prepared an aptasensor by modifying a glassy carbon electrode with a MWCNTs-AuNPs/CS-AuNPs/rGO-AuNPs nanocomposite, followed by binding with an amine-modified aptamer [19]. Yang et al. have developed an electrochemical aptasensor by covalently binding an *OTC* aptamer on a glassy carbon electrode via a diazonium coupling reaction [20]. In a separate study, a Ce-MOF@COF hybrid nanostructure was immobilized on a gold electrode, followed by further binding of an *OTC*-specific aptamer [21]. Wang et al. have reported on a HOF-based electrochemical *OTC* aptasensor in which a gold electrode was modified sequentially with a HOF and an *OTC* aptamer [22]. Finally, another electrochemical aptasensor for *OTC* was fabricated after modification of a glassy carbon electrode with a rGO–ZnO–Au nanocomposite and incubation with a SH-modified aptamer [4]. However, all these aptasensors have some drawbacks (Table S1, Supplementary Material); they utilize gold or carbon macroelectrodes and require bulky external reference and counter electrodes, therefore they are limited to the analysis of larger volumes of samples, therefore are unsuitable for on-site assays; in addition, these methods are based on nanomaterial-modified electrodes, but the preparation of nanomaterials is, generally, laborious and time-consuming; and finally, no provision or study is made to specifically address the issue of matrix effects which are expected to be significant in milk samples. Recently, our group described a microfabricated electrochemical aptasensing transducer with antifouling properties fabricated by gold sputtering [23]. Sputtering is a mature thin-film deposition technology that enables the fabrication of miniaturized electrodes that can be integrated into a portable device, offers increased scope for mass fabrication, allows for the deposition of highly pure and uniform thin films as well as for precise control over the thickness of the deposited material [24]. This preliminary work demonstrated proof-of-principle applicability to *OTC* detection, but the sensor still required external reference and counter electrodes; it utilized an aptamer that was not fully characterized; the detection protocol was not optimized; and the methodology was not validated with application to samples.

In this work, we have extended this concept by developing an integrated 3-electrode microfabricated electrochemical aptasensing device for single-drop label-free assay of *OTC* in milk. The integrated chip consists of gold working and counter electrodes and a silver reference electrode deposited by sputtering of the respective metals on a Kapton film. The working electrode is modified with *α*-lipoic acid-NHS—which forms a self-assembled monolayer on the working electrode surface and serves as both an antifouling compound and as a linker—onto which an amine-terminated *OTC*-specific aptamer is immobilized via covalent bonding. The preparation and the analytical performance of the aptasensor are studied by different electrochemical and optical techniques.

## Materials and methods

### Chemicals and reagents

Reagents and chemicals were of analytical grade, provided by Merck (Darmstadt, Germany) and Sigma-Aldrich (Burlington, MA, USA), and used with no further purification.

The base sequence of the selected aptamer was *OTC*-APT5: 5′-ACG ACA TTC CGT TGA TCT CTC CCT TTT GGG TTG GTG TCG T-3′. Its thiol-terminated version was 5′-ACG ACA TTC CGT TGA TCT CTC CCT TTT GGG TTG GTG TCG T/3ThioMC3-D/−3′; its amine-terminated version was 5′-ACG ACA TTC CGT TGA TCT CTC CCT TTT GGG TTG GTG TCG T/3AmMO/−3′; these aptamers were purchased from Integrated DNA Technologies Inc. (Coralville, IA, USA). The amine-terminated and fluorescein-labeled version was 5′-FAM-ACG ACA TTC CGT TGA TCT CTC CCT TTT GGG TTG GTG TCG T/3AmMO/−3′ from Microsynth (Balgach, Switzerland).

The antifouling linker, *α*-lipoic acid-NHS, was purchased from MedChemExpress (Monmouth Junction, NJ, USA), and *α*-lipoic acid was purchased from Merck (Darmstadt, Germany). A 2 mM stock solution of both compounds was prepared in EtOH 50% (v/v).

The ELISA *OTC* kit was obtained from CD creative Diagnostics (Shirley, NY, USA).

Stock solutions (100 μM) of the aptamers were prepared in nuclease-free molecular water and stored at − 19 °C. For the preparation of the working aptamer solutions, a volume of the stock solution was heated at 95 °C in a water bath for 5 min. Then, the aptamer was let to cool at room temperature. For the electrochemical assays, more dilute solutions were prepared in phosphate buffer (PB) (10 mM, pH 7.4). For the CD and fluorescence experiments more dilute solutions were prepared in a solution containing 20 mM tris, 100 mM NaCl, 5 mM KCl, 2 mM MgCl_2_, 2 mM CaCl_2_, and 0.05% Tween 20.

Oxytetracycline (*OTC*) hydrochloride salt was purchased from Thermo Fisher Scientific (Waltham, MA, USA). For the electrochemical assays, a 100 mg L^−1^ *OTC* stock solution was prepared in water, and more dilute *OTC* standard solutions were prepared in PB (10 mM, pH 7.4) containing 2 mM MgCl_2_. For the CD and fluorescence experiments a 8 mM *OTC* stock solution was prepared in water, and more dilute solutions were prepared in a solution containing 20 mM tris, 100 mM NaCl, 5 mM KCl, 2 mM MgCl_2_, 2 mM CaCl_2_, and 0.05% Tween 20.

Flexible Kapton® HN polyimide film (50-μm thicknesses) was purchased from RS components (RS, Corby, UK). The Cr, Au, and Ag sputtering targets (99.999% purity) were purchased from Kurt Lesker (East Sussex, UK).

For the experiments in milk, 0.1 g of low-fat dried milk powder was dissolved in 10 mL or 50 mL PB (10 mM, pH 7.4), containing 2 mM MgCl_2_, and centrifugated for 15 min at 950 rpm. The supernatant solution was used for spiking with *OTC*.

### Fabrication of the sensor chips

**Scheme 1:**
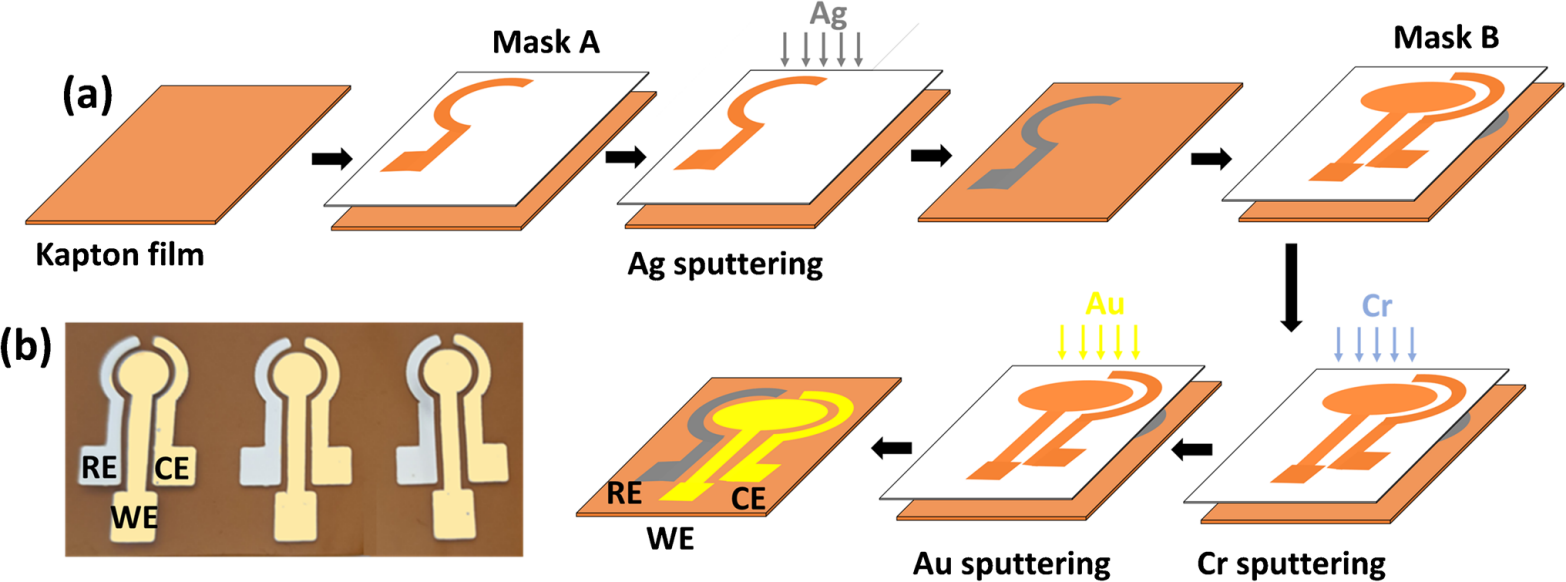
**a** Schematic of the fabrication procedure of the sensor chip and **b** photo of 3 chips

The integrated three-electrode chips are fabricated via DC magnetron sputtering, as illustrated in Scheme 1 (a). Deposition was performed under a vacuum at 3 mTorr. A film of flexible Kapton® HN polyimide film was covered with a metal mask (Mask A) with the pattern for the reference electrode (RE), and Ag was sputtered at a thickness of 100 nm (power density 0.5 W/cm^2^ and deposition rate 0.12 nm/s). Then, a second mask (Mask B), with the pattern of the counter electrode (CE) and the working electrode (WE), was properly aligned on the Kapton film and Cr and Au were sputtered on the film at nominal thicknesses of 5 nm (power density 3 W/cm^2^ and deposition density 0.28 nm/s) and 100 nm (power density 1.5 W/cm^2^ and deposition density 0.4 nm/s), respectively.

A photo of 3 chips is illustrated in Scheme 1 (b), while the nominal dimensions of the chip components are shown in Figure S1 (Supplementary Information). Before an electrochemical experiment, the working electrode was activated by performing 5 cyclic voltammetry (CV) cycles in the potential range − 1.0 V to + 0.6 V in in 0.1 M H_2_SO_4_.

### Instrumentation

A PGSTAT12 Autolab potentiostat equipped with the GPES 4.9 Software (Metrohm, Herisau, Switzerland) was used for the electrochemical measurements. The measurement conditions for the anodic differential pulse voltammetry (DPV) were as follows: − 0.15 to + 0.45 V, scan rate 10 mV/s, step 5 mV, pulse amplitude 25 mV, modulation time 50 ms, and interval time 0.5 s. The measurement conditions for the CV were as follows: − 0.4 to + 0.6 V and scan rate 100 mV/s. For the electrochemical impedance spectroscopy (EIS), the measurement conditions were as follows: DC potential + 0.25 V, AC potential 10 mV, and frequency range 100,000 Hz to 0.1 Hz.

Circular dichroism (CD) spectra were recorded (200–320 nm range, 2 nm bandwidth, and 50 nm/min scan rate) using a Jasco J-1500 CD spectrometer (Tokyo, Japan) thermostated at 25 °C. For the fluorescence titrations, a Tecan Spark® Multimode Microplate Reader (Männedorf, Switzerland) was used.

Sputtering was performed with a CV401 system (Cooke Vacuum Products, W. Redding, CT, USA) using metal masks to define the sputtering area. An atomic force microscope (AFM) (SPM SMENA, NT-MDT Co) and an XRD spectrometer (Siemens D500, Bruker GmbH) using the Ni-filtered Cu-Ka radiation line were used for characterizing the Au working electrode.

Α UV lamp with emission at 254 nm (model UV-8 S/L, Herolab, Germany) was used as an excitation source in fluorescence measurements. The fluorescent images were captured with a smartphone camera (Samsung A33).

### Fluorescence, CD spectra, and fluorescence titrations

The experimental details and procedures for studying the binding of the aptamer to the linker with fluorescence and for recording the CD specta and the fluorescence titrations are described in the Supplementary Material.

### Preparation of the aptasensor

**Scheme 2:**
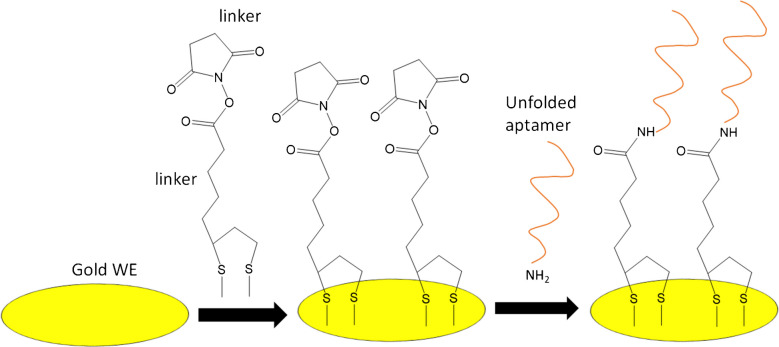
Schematic diagram of the fabrication of the aptasensor

The preparation of the aptasensor is schematically illustrated in Scheme 2. Twenty-five microliters of a 2 mM *α*-lipoic acid-NHS solution was drop-casted on the gold WE of the sensor chip and incubated for 12 h at 4 °C in a humidity chamber in order to form a self-assembled monolayer of the unfolded aptamer on the surface via the strong Au–S covalent bonds [25]. Then, the sensor was rinsed thoroughly with deionized water and dried under a gentle nitrogen stream. Twenty-five microliters of a 4 μM of the amine-modified aptamer solution was drop-casted on the working electrode surface and left for 2.5 h at room temperature to form the respective amide [26]. Then, the electrode was washed thoroughly with deionized water to remove any excess aptamer molecules and air-dried. The aptasensors were stored at 4 °C in sealed bags.

### Aptamer-based assay

**Scheme 3:**
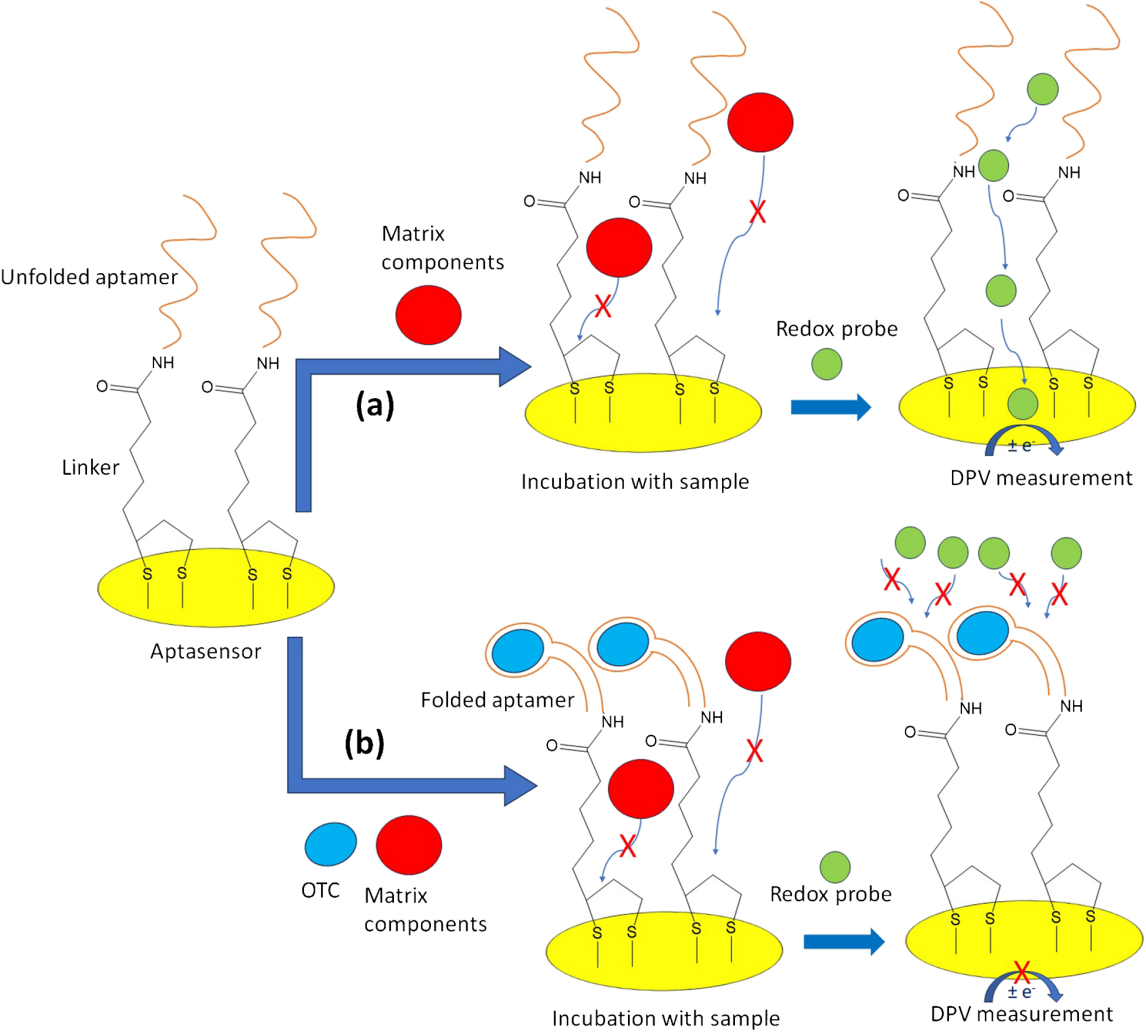
Schematic diagram of the aptamer-based electrochemical assay **a** in the absence of *OTC* and **b** in the presence of *OTC*

The assay was performed by adding a 25 μL drop of sample or *OTC* standard solution on the WE of the aptasensor and incubating for 30 min. Then, the WE was washed with deionized water. Finally, the anodic DPV response at the aptasensor was obtained after adding 60 μL of a 10 mM Fe(CN)_6_^4−^/Fe(CN)_6_^3−^ probe solution in 0.5 M KCl making sure that the solution covers the 3 electrodes of the chip. In the case that no *OTC* was present in the sample, the aptamer remained in the unfolded configuration which allowed the diffusion of the probe to the WE surface, generating a strong current (Scheme 3(a)). The presence *OTC* induced a conformational change (folding) inhibiting the diffusion of the probe to the WE surface resulting in a weaker current (Scheme 3(b)). During the incubation period with the sample, the antifouling linker *α*-lipoic acid-NHS prevented adsorption of the sample matrix components on the WE of the sensor (Scheme 3(a) and Scheme 3(b)).

### Data evaluation

The analytical signal was expressed as the % relative anodic DPV current reduction, *I*%, calculated as *I*% = (*i*_*s*_* − i*_*o*_)/*i*_*o*_ × 100 (where *i*_*o*_ is the anodic DPV peak current of the redox probe at the aptasensor before incubation with *OTC*, and *i*_*s*_ is the anodic DPV peak current at the aptasensor after incubation with *OTC*).

## Results and discussion

### Characterization of the aptamer-OTC binding

**Fig. 1:**
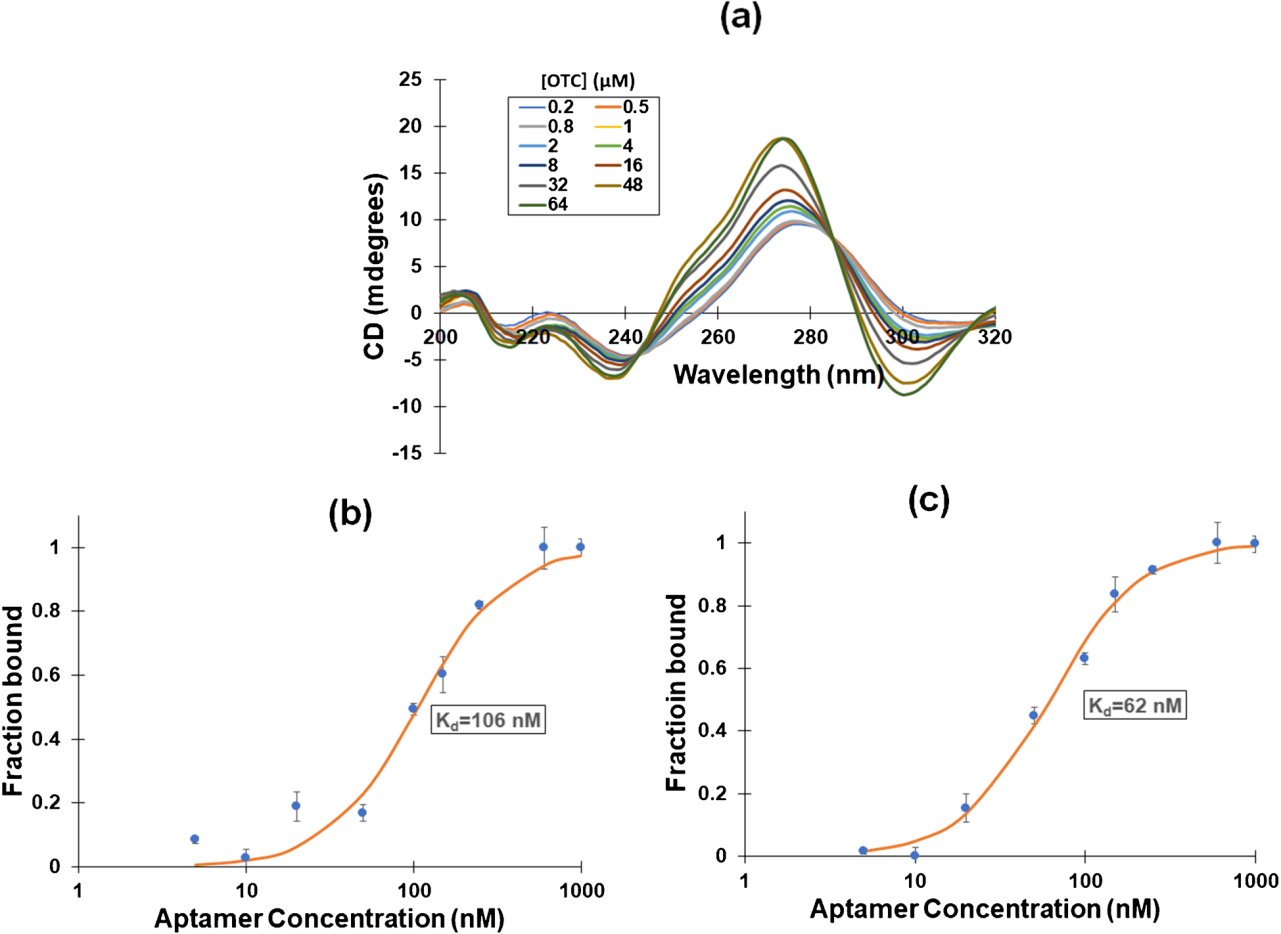
**a** CD spectra of solutions containing 2 μΜ of the aptamer and increasing concentrations of *OTC*. **b** Fluorescence and **c **fluorescence polarization titration plots in a solution containing 200 nM *OTC* and increasing concentrations of the aptamer used for the determination of K_d_

Various *OTC*-specific aptamers have been reported in the literature [27–31]. Our preliminary experiments have shown that the aptamer introduced by Zao et al. [29] produced the most promising results and was selected for the rest of this study. The aptamer-*OTC* binding was studied by using CD spectroscopy [32]. The appearance of a negative peak at c.a. 240 nm and a positive peak at c.a. 275 nm suggests that the aptamer structure adopts a B-form. Upon addition of *OTC*, a peak at c.a. 300 nm appears indicating a possible B to Z structural transition. Also, clear changes in the peak magnitude at c.a. 275 and 300 nm of the aptamer spectrum were observed upon addition of *OTC* (Fig. 1(a)), from which it can be deduced that binding does occur and that the aptamer is suitable for analytical purposes [33]. This assumption is reinforced by binding studies of *OTC* with the aptamer using fluorescence and fluorescence polarization measurements [34, 35]. Sigmoidal titration curves for both fluorescence and fluorescence polarization were obtained, from which the equilibrium dissociation constant (*K*_*d*_) was calculated as 106 mM and 62 nM, respectively (Fig. 1(b) and (c)) [36]. These values are of the same order of magnitude as measured in previous studies (150 nM using fluorescence [27] and 107 nM using isothermal titration [30] but in different buffers).

### Characterization of the sensor chip

**Fig. 2:**
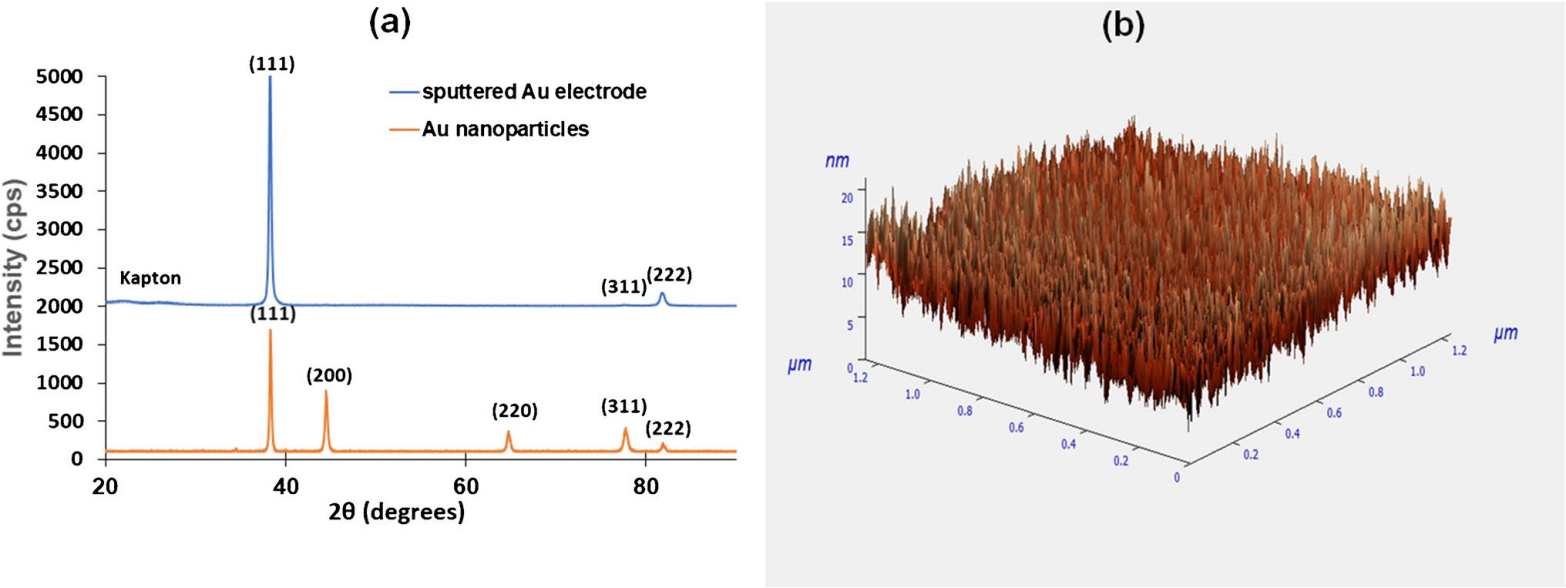
**a** XRD spectra of the sputtered gold WE and gold nanoparticles and **b** AFM of the surface of the WE

The surface of the working electrode of the sensor chip was studied by XRD and AFM (Fig. 2). The XRD spectrum of the gold thin-film electrode shows an intense Au peak at a 2*θ* value of 38.1°, suggesting a dominant [111] crystalline orientation, followed by a weaker Au (311) peak at a 2*θ* value of 77.1° and an Au [222] peak at a 2*θ* value of 81.7°. The contribution of the Kapton film can be observed in the wide signal at 2*θ* in the 20–30° range, while no visible peak was obtained from the buffer Cr film due to its low thickness (5 nm). Therefore, the deposition of gold on Kapton exhibited an essentially monostrystalline structure, as opposed to nanoparticle-modified electrodes that typically display a more pronounced polycrystalline surface morphology (Fig. 2(a)). The AFM imaging of the thin-film gold electrodes indicates that the gold film is exceptionally smooth with a roughness of c.a. 2.5 nm and a grain diameter of c.a. 15 nm (Fig. 2(b)). This smooth surface morphology, which is suitable for the reproducible and uniform attachment of the aptamer, as opposed to the commonly used thick-film screen-printed gold deposits that exhibit a much rougher surface (with average roughness in the order of 1 μm), and, consequently, lower device-to-device uniformity [37, 38]. In addition, sputtering does not require any post-fabrication treatment (curing), as is the case with screen-printing and allows for better fabrication resolution. Although sputtering requires clean-room facilities and dedicated instrumentation, it enables large-scale fabrication resulting in reduced manufacturing costs per sensor.

The medium-term stability of the Ag reference is paramount, since each sensor must undergo long incubation periods. The drift of the reference electrode potential was studied at six chips for a period of 24 h with respect to a commercial Ag/AgCl electrode in a 0.1 mol L^−1^ KCl solution; the potentials of the reference electrodes remained with ± 5% of their mean values indicating satisfactory medium-term drift. The repeatability between the potentials of the reference electrodes of the six chips was measured after 24 h of their immersion in in a 0.1 mol L^−1^ KCl solution; the coefficient of variation of the potentials was 7.3%, suggesting sufficient reference electrode reproducibility between chips.

Then, the activation of the WE was investigated by CV in a 10 mM Fe(CN)_6_^4−^/Fe(CN)_6_^3−^ solution in 0.5 M KCl (Figure S3, Supplementary Material). It was found that, without activation, the electrochemical response was poor, and the peak separation in the CV was 590 mV, indicating an irreversible redox reaction with sluggish kinetics (Figure S2, Supplementary Material) [39]. Although activation of gold electrodes is normally carried out by CV scanning up to positive potentials in acidic solution involving repetitive cycles of oxidation of gold to gold oxide and reduction of the oxide, this method may lead to restructuring or roughening of the surface. Therefore, a “mild activation” protocol was used by scanning up to an anodic limit of + 0.6 V in 0.1 M H_2_SO_4_ [40]. After activation by running 5 CV scans from − 1.0 V to + 0.6 V, the performance of the WE was improved dramatically as the magnitude of both the reduction and oxidation peaks of the Fe(CN)_6_^4−^/Fe(CN)_6_^3−^ couple increased significantly, and the peak separation was reduced to 114 mV, indicating a quasi-reversible redox process with improved electrode kinetics (Figure S2, Supplementary Material) [39].

### Study of the aptasensor preparation with electrochemical techniques

**Fig. 3:**
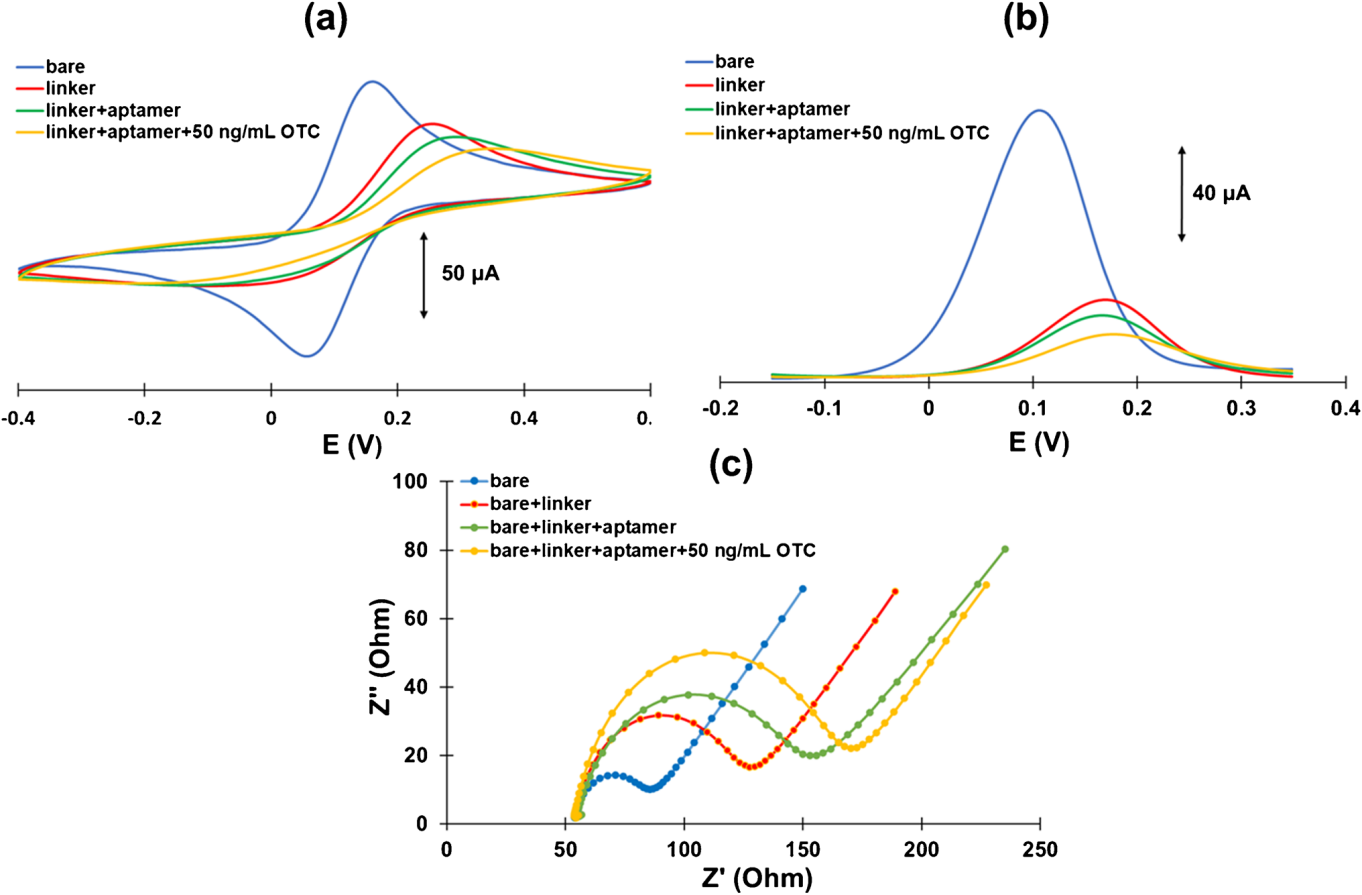
**a** CV, **b** anodic DPV, and **c** EIS measurements in a solution containing 10 mM Fe(CN)_6_^4−^/Fe(CN)_6_^3−^ solution in 0.5 M KCl taken at the different stages of the aptasensor preparation

CV, DPV, and EIS were employed to study the changes in the electrochemical response of the [Fe(CN)_6_]^4−^/[Fe(CN)_6_]3^−^ redox probe at different stages of the aptasensor preparation, and to verify the binding of *OTC*; typical measurements are presented in Fig. 3 (a), (b), and (c), respectively.

The CV scans (Fig. 3(a)) indicated large anodic and cathodic peaks at the bare WE which gradually decreased after immobilization of the linker and of the aptamer. In addition to the signal decrease, the peak separation between the anodic and cathodic peaks increased, suggesting slower reaction kinetics as a result of the successive modification layers on the surface of the WE. An addition of *OTC* caused a further decrease of the redox peaks, demonstrating that the presence of the *OTC* caused a conformational change that induced an associated reduction in the response.

Regarding the anodic DPV (Fig. 3(b)), the results showed the same pattern as the CVs with a gradual decrease of the response after the immobilization of the linker, the incubation with aptamer, and the binding with *OTC*. The peak potentials were also shifted to more anodic values as the oxidation of the probe required an additional overpotential to occur at the modified electrodes. It was noteworthy that the signal changes in DPV were generally more pronounced and well-defined than in CV. For this reason, anodic DPV was used for further experiments and for analytical purposes.

The EIS spectra (Fig. 3(c)) corroborated these data, demonstrating a gradual increase in the charge–transfer resistance after immobilization of the linker, the aptamer, and *OTC* on the WE.

### Study of the chemical and instrumental parameters

**Fig. 4:**
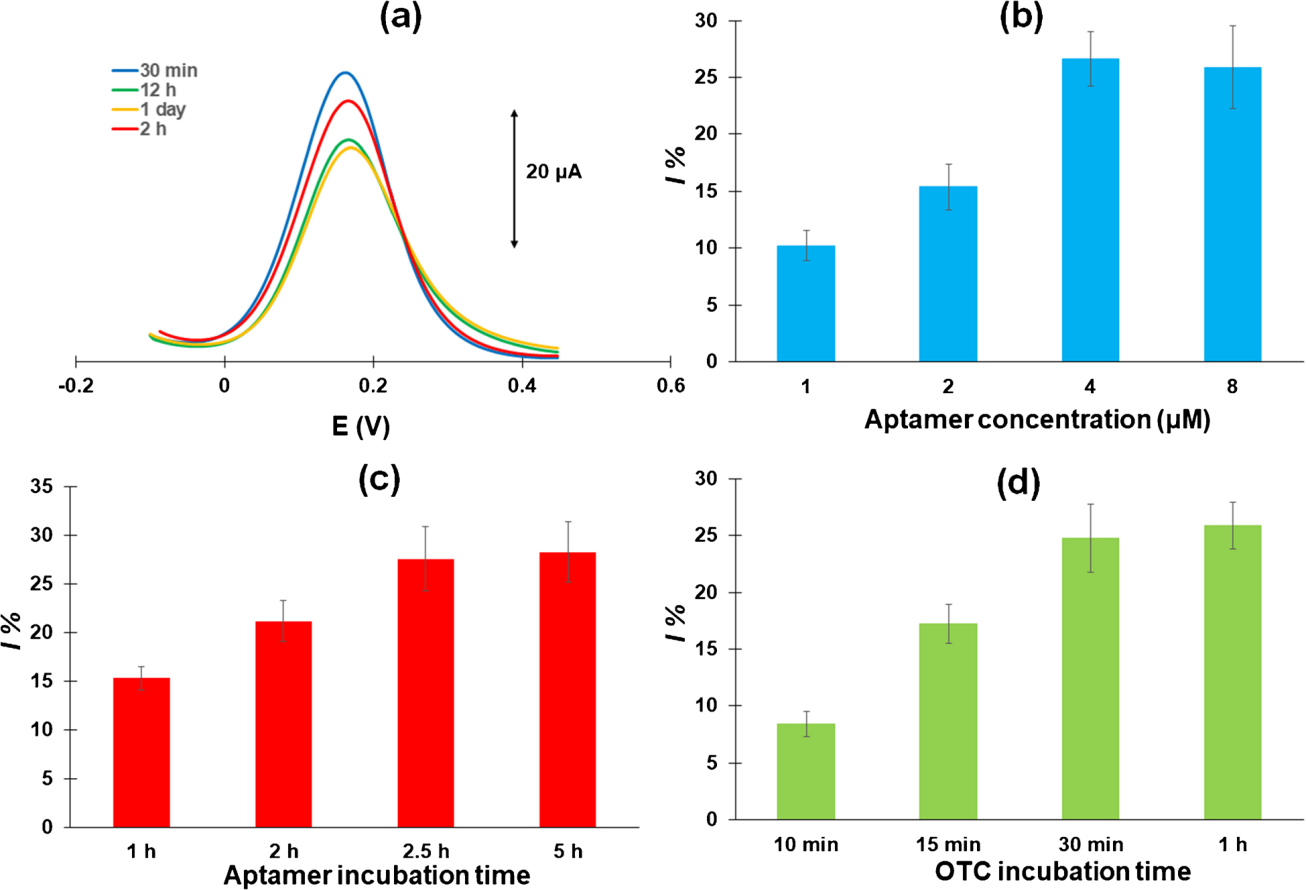
Effect of **a** the linker incubation time, **b** the aptamer concentration, **c** the aptamer incubation time, and **d** the *OTC* incubation time in a solution containing 50 ng mL^−1^*OTC*

As a first step in the construction of the aptasensor, an effective SAM of the linker should be formed at the surface of the WE. The linker contains a disulfide bond S–S which by itself is effective in forming SAMs on gold surfaces [39–42]. Comparative experiments using tris(2-carboxyethyl)phosphine (TCEP) for the reduction of the S–S bonds before attachment of the linker on the gold WE produced similar or worse results than using the untreated linker. The formation process of dithiols SAMs on gold has been postulated as occurring in two steps [41–44]: disulfides first physisorbs rapidly on gold in a random configuration. Then, a slower reorganization step takes place to a “standing up” ordered conformation perpendicular to the surface (as schematically illustrated in Scheme 2). Therefore, an incubation time of more than 2 h has been recommended to achieve successful SAMs [44]. In this work, the incubation time was varied between 30 min and 1 day, and the anodic DPVs were recorded in the redox probe solution. The results are summarized in Fig. 4 (a), indicating that after 12 h of incubation, the linker film has achieved its final spatial configuration, since no further change in the anodic DPV response was observed which is in agreement with another study involving *α*-lipoic acid immobilization on gold [45]. The binding of the aptamer to the linker was studied via fluorescence experiments using an aptamer modified with fluorescein (at the 5′-end) and amine (at the 3′-end), as illustrated in Figure S3 (Supplementary Material). When the WE of the sensor was modified with *α*-lipoic acid (without the NHS moiety) and the fluorescein/amine-modified aptamer, only weak fluorescent patches were observed after rinsing (Figure S3(a)). This is attributed to the fact that most of the aptamer was removed by rinsing and is indicative of weak attachment of the aptamer on the surface of the WE. In contrast, when the WE of the sensor was modified with *α*-lipoic acid-NHS and the fluorescein/amine-modified aptamer, a strong and uniformly distributed fluorescence intensity was recorded after rinsing, showing that the aptamer was strongly bound across the entire surface of the electrode (Figure S3(b)). To preclude the possibility that the aptamer binds directly to the gold surface in addition to the linker, the WE of the sensor was modified only with the amine/fluorescein modified aptamer without prior modification with the linker; after rinsing of the WE, no fluorescence was observed (Figure S3(c)), demonstrating that the aptamer does not attach to the bare electrode surface. These results support the assumption that the *α*-lipoic acid-NHS linker facilitates the attachment of the aptamer of the surface, presumably via covalent bonding.

Next, the concentration of the aptamer incubated with the *α*-lipoic acid-modified WE was studied in the range of 2–8 μΜ. As illustrated in Fig. 4 (b), the signal intensity increased with increasing aptamer concentration and it stabilized at 4 μΜ of aptamer which was selected for the following experiments. This can be accounted for by assuming that at lower concentrations of aptamer, some binding sites were not covered by aptamer leading to lower sensitivity, while at higher concentration of the aptamer, all the binding sites were occupied by the aptamer.

The time of incubation between the aptamer and the *α*-lipoic acid-modified WE was studied in the range of 30 min to 5 h. As illustrated in Fig. 4 (c), the signal intensity increased with increasing incubation time up to 2.5 h which was used for further experiments.

The time of incubation between the sample containing *OTC* and the aptasensor was studied in the range of 5 min to 1 h. As illustrated in Fig. 4 (d), the signal intensity increased with increasing incubation time up to 30 min which was used for further experiments.

The effect of the sample pH was studied by measuring the signal of a 50 ng mL^−1^ *OTC* standard solution prepared in phosphate buffers (PB) in the pH range 6.2–7.8 (also containing 2 mM MgCl_2_), and no statistically significant differentiation of the signal was observed within this pH range.

### Analytical and operational features-matrix effects

**Fig. 5:**
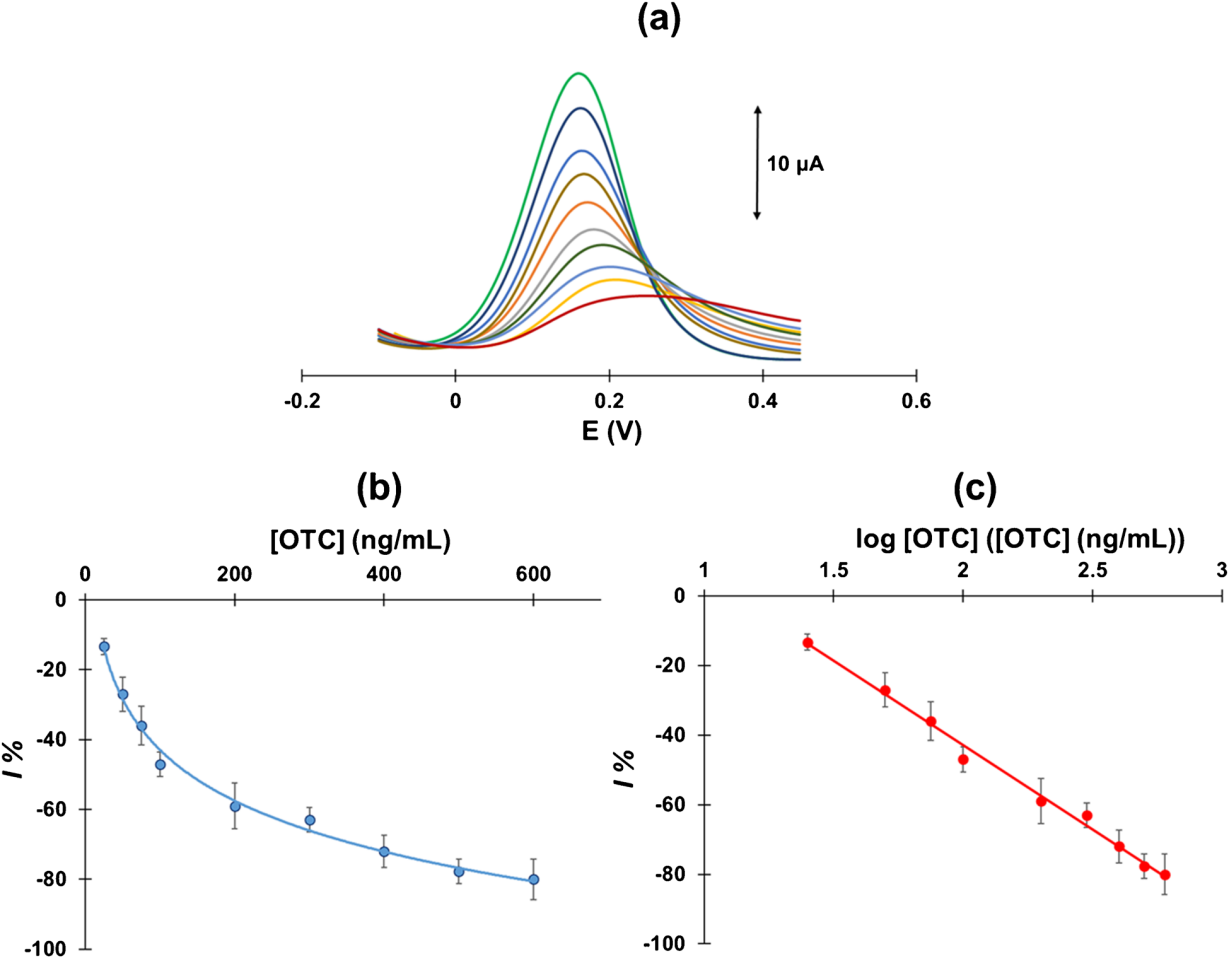
**a** Anodic DPV traces in standard *OTC* solutions covering the range 25– 600 ng L^−1^, **b** calibration plot of *I*% vs. *OTC* concentration, and **c** linearized calibration plot of *I*% vs. the logarithm of *OTC* concentration

Calibration for *OTC* was performed by anodic DPV in the concentration range 0–600 μg L^−1^*.* Typical anodic DPV traces are illustrated in Fig. 5 (a), showing that the anodic DPV peaks decrease in height, gradually shift to more anodic potentials, and become wider as the *OTC* concentration increases. These features are indicative of less favorable diffusion/oxidation process induced by the presence of increasing concentrations of *OTC*. The calibration curve was plotted as *I*% vs. the *OTC* concentration with each point being the average of three measurements (Fig. 5(b)). It should be noted that using the % relative signal reduction (*I*% = (*i*_*s*_* − i*_*o*_)/*i*_*o*_ × 100), rather than the absolute value of the signal reduction, (*i*_*s*_* − i*_*o*_), has the advantage that the blank signal serves as an in-built internal standard which corrects for variations between different sensors. The calibration plot was linearized by plotting *I*% vs. log *OTC* concentration (Fig. 5(c)) with a coefficient of determination *R*^2^ = 0.993. The limit of detection (*LOD*) was calculated as 7 ng mL^−1^ using the formula *LOD* = 3.3 × s/S (where *s* is the standard deviation of the intercept of the linear plot of (*i*_*s*_* − i*_*o*_) vs. the *OTC* concentration at the three lowest concentrations (25, 50, and 75 ng mL^−1^), and *S* is the slope of the same plot) [46]. The average between-sensors repeatability across the whole calibration range was 13.7%. The shelf-time of the aptasensors was estimated by preparing 6 identical aptasensors and measuring the signal of a 50 ng mL^−1^*OTC* standard solution in a period of 16 days using a different sensor every 3 days. As illustrated in the control chart (Figure S4, Supplementary Material), the signal exhibited a gradual decrease with time but remained within the standard deviation of the methodology. Therefore, it could be considered that the aptasensors could be reliably used for a fortnight after preparation.

In this work, special attention was focused on the effect of matrix effects due to non-specific absorption of the sample components on the WE that can cause fouling of its surface. This effect has been recognized as a persistent challenge in biosensing applications in complex food and clinical samples, thus different strategies have been proposed to alleviate it [47–50]. Among them, SAMs of alkanethiols and polyethylene-glycol derivatives are widely used as antifouling coatings because they are easy to immobilize, are inexpensive, and their structure can be tailored [51, 52]. The Thompson group has made significant advances in synthesizing and successfully applying several SAM linkers that serve a dual role as both antifouling agents and as anchors to covalently attach aptamers, thereby creating smart interfaces for both biorecognition and alleviation of electrode fouling [53–55]. However, synthesis of these compounds is time-consuming and laborious, while further modification with NHS chemistry is required for attachment to amine-modified aptamers. Recently, a new linker, *α*-lipoic acid-NHS, with antifouling properties has been proposed [23]. This compound is commercially available, can directly bind to amine-modified aptamers, and is able to form SAMs on gold surfaces [23, 45].

**Fig. 6:**
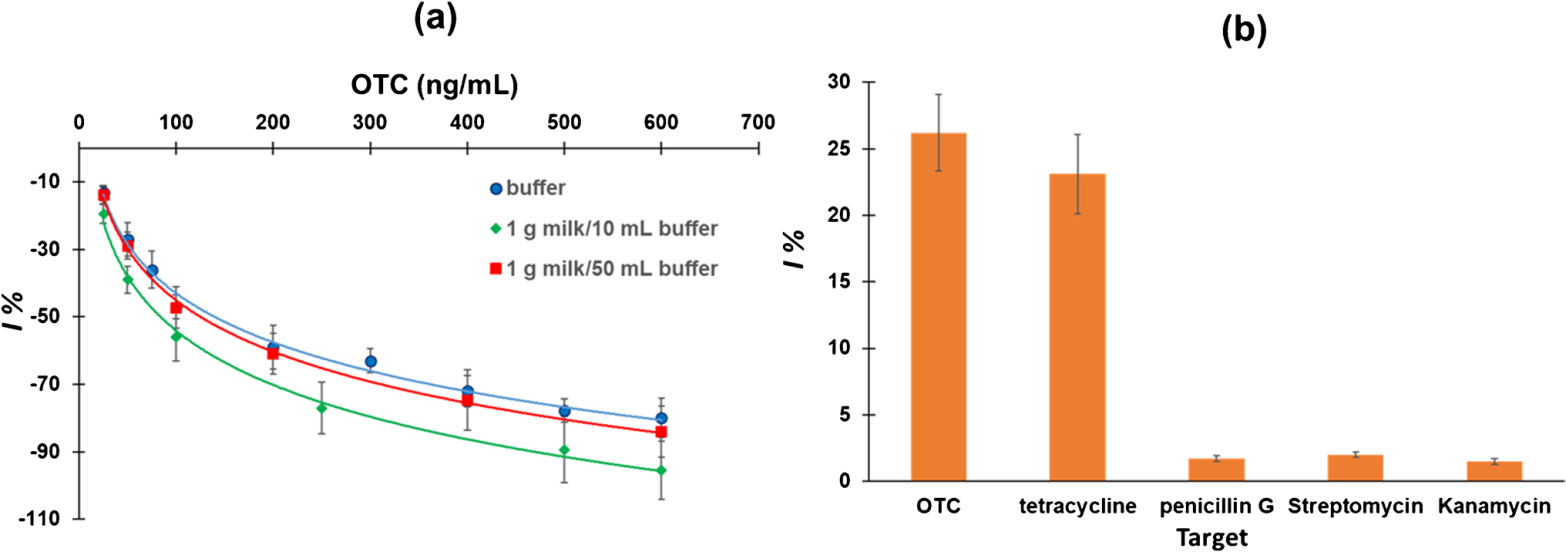
**a** *OTC* calibration plots in buffer and in milk using 2 dilution factors, **b** specificity of the aptasensor in the presence of 50 ng mL^−1^ *OTC* and tetracycline, and 250 ng mL^−1^ penicillin G, streptomycin, and kanamycin

The antifouling properties of *α*-lipoic acid-NHS in milk were investigated by performing comparative anodic DPV measurements in buffer solution and in milk samples before and after addition of *OTC* at two milk dilutions (1 g milk/50 mL buffer and 1 g milk/10 mL buffer). For both the milk dilutions, the magnitude of the DPV peak heights in the absence and presence of *OTC* were reduced in the milk solution, compared to the buffer. In addition, for the lower milk dilution (1 g milk/10 mL buffer), the *I*% was also significantly lower than in the buffer indicating a significant matrix effect (Fig. 6(a)). In contrast, for the higher milk dilution (1 g milk/50 mL buffer), the *I*% did not statistically differ from that in the buffer across the whole calibration range of 25–600 ng mL^−1^*OTC* (Fig. 6(a)). Therefore, using a higher dilution can compensate for the matrix effect and the calibration plot in buffer could be used for quantification. In order to further demonstrate the utility of the antifouling linker, comparative measurements of aptasensors with the same aptamer in the presence and in the absence of the linker were performed. Since the amine-modified aptamer could not be immobilized efficiently on the gold WE, an aptasensor was prepared involving the immobilization of the thiol-modified version of the same aptamer on the WE surface using an existing protocol without the use of linker [56]. As illustrated in Figure S5 (Supplementary Material), the presence of the milk matrix, even for the more diluted milk sample, almost completely suppressed the anodic DPV current of the aptasensor without the linker. This suggests that, while the use of α-lipoic acid-NHS may not entirely alleviate matrix effects, it does play a significant role in enabling the production of a quantifiable current signal, and its use allows for detection in milk samples.

### Specificity

Various antibiotics (ampicillin, penicillin G, tetracycline, kanamycin, and streptomycin) were tested as potential interfering species with the determination of *OTC*. As illustrated in Fig. 6 (b), ampicillin, penicillin G, kanamycin, and streptomycin at a fivefold excess over *OTC* cause a change in *I*% lower than 10% with respect to the signal of *OTC*. The aptamer, being a group-specific aptamer, binds tetracyclines, and a change in *I*% that did not statistically differ from the signal of *OTC* was induced by the presence of similar concentration of tetracycline [27, 29, 30]. Therefore, the aptasensor could potentially be used for semi-quantitative detection of total tetracyclines.

### Applications

The method using the integrated aptasensensing chip was applied to the determination of a diluted powdered milk sample (1 g milk/50 mL buffer) spiked with *OTC* at two different concentrations. The respective anodic DPVs are illustrated in Figure S6 (Supplementary Material), and the results are summarized in Table 1 and compared to those obtained with a commercial ELISA kit for *OTC*; a *t*-test indicates that the two methods produce results that do not statistically differ at the 95% confidence level. The recommended reconstitution of the powdered milk is ~ 1 g milk/10 mL of water, meaning that the sample used for the analysis was diluted five-fold with respect to the recommended reconstitution. Given that the *LOD* of the method was 7 ng mL^−1^ using the diluted powdered milk sample (1 g milk/50 mL buffer), 35 ng mL^−1^ could be detected in the reconstituted sample which is lower than the 100 ng mL^−1^ MRL set by the EU. Therefore, the method is fit-for-purpose for the determination of *OTC* in milk.

**Table 1:** Results for the determination of *OTC* in spiked milk samples

*OTC* added (ng mL^−1^)	*OTC* determined (ng mL^−1^)		Recovery (*RSD*%)	
	Aptasensor	ELISA	Aptasensor	ELISA
50	55	48	110 (15)	96 (11)
300	320	315	107 (13)	105 (9)

## Conclusions

In this work, a microfabricated electrochemical aptasensing chip was developed for label-free assay of *OTC* in milk. The electrodes of the sensor were fabricated through a metal sputtering process that lends itself to large-scale production. The gold working electrode of the sensor was further modified with a dual-function antifouling compound which endows the aptasensor with antifouling properties and serves as a linker for covalent attachment of the *OTC*-specific aptamer without any chemical modification. The *LOD* achieved in reconstituted milk samples is lower than the *MRL* set by the EU. As a result of the combination of these innovations, this aptasensor is self-contained and portable; its fabrication is simple and highly reproducible and can operate in a drop-volume label-free mode in milk with good tolerance against matrix effects.

## Supplementary Information

Below is the link to the electronic supplementary material.

ESM 1 (DOCX 341 KB)

## Data Availability

Data is provided within the manuscript or supplementary information files.
