# Supplementary material for: Microfabricated electrochemical aptasensing chip modified with dual-function antifouling linker for single-drop label-free assay of oxytetracycline in milk
Source: Mikrochim Acta. 2025 Jul 28;192(8):527. doi: 10.1007/s00604-025-07387-4 (PMC12304045; doi:10.1007/s00604-025-07387-4)
Supplement: Supplementary file 1 — (DOCX 341 KB) [file 604_2025_7387_MOESM1_ESM.docx]

**Supporting Information**

Microfabricated electrochemical aptasensing chip modified with dual-function antifouling linker for single-drop label-free assay of oxytetracycline in milk

Christina Bizindi^1^, Dionysios Soulis^1^, Dimitra Kourti^1^, Georgia Geka^1^, Christos Kokkinos^1^, Michael Thompson^2^, Thanassis Speliotis^3^, Anastasios Economou^1^

*^1^ Department of Chemistry, National and Kapodistrian University of Athens, Athens 157 71, Greece*

*^2^ Department of Chemistry, University of Toronto, 80 St. George St., Toronto, Ontario, M5S 3H6, Canada*

*^3^ Institute of Nanoscience and Nanotechnology, N.C.S.R. Demokritos, P.O. Box 60037, 15310 Agia Paraskevi, Greece*

**Table S1**. Existing applications of electrochemical label-free aptasensors for OTC.

| Working electrode | Integration | Electrode modification | Study of matrix effects/antifouling agent | Scope for on-site analysis | LOD | Reference |
| --- | --- | --- | --- | --- | --- | --- |
| glassy carbon | No | Floral ZnO/rGO/Colloidal Au composite | No/no | No | 0.33 μg L^-1^ | [4] |
| glassy carbon | No | rGO, MWCNTs, chitosan, AuNPs | No/no | No | 0.03 μg L^-1^ | [19] |
| glassy carbon | No | 4-carboxyphenyl diazonium salt | No/no | No | 0.33 μg L^-1^ | [20] |
| Bulk gold | No | Ce-MOF@MCA | No/no | No | 0.03 μg L^-1^ | [21] |
| Bulk gold | No | HOFs | No/no |  | 0.001 μg L^-1^ | [22] |
| Sputtered gold | Yes | *α*-lipoic acid-NHS | Yes/*α*-lipoic acid-NHS | Yes | 7 μg L^-1^ | This work |

rGO, reduced graphene oxide; MWCNTs, multi-walled carbon nanotubes; AuNPs, gold nanoparticles; MOF, molecular imprinted polymer; MCA, melamine-cyanuric acid nanohybrid; HOFs, hydrogen organic frameworks


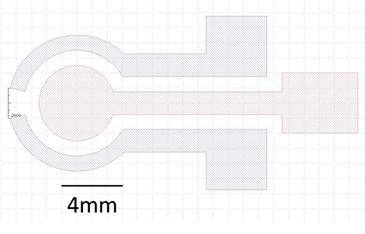


**Figure S1.** Nominal dimensions of the sensor chip.

##

**Study of the linker aptamer-linker binding with fluorescence experiments**

In order to study the binding of the linker to the aptamer, the working electrodes of 3 sensor chips were modified as follows:

**(a):** 25 μL of a 2 mM *α*-lipoic acid solution was drop-casted on the gold WE of the sensor chip and incubated for 12 h at 4 °C in a humidity chamber. Then, the sensor was rinsed thoroughly with deionized water and dried under a gentle nitrogen stream. 25 μL of a 4 μM fluorescein/amine-modified aptamer solution (5’-FAM-ACG ACA TTC CGT TGA TCT CTC CCT TTT GGG TTG GTG TCG T/3AmMO/-3′) was drop-casted on the working electrode surface and left for 2.5 h at room temperature. Then, the electrode was washed thoroughly with deionized water and air-dried.

**(b):** 25 μL of a 2 mM *α*-lipoic acid–NHS solution was drop-casted on the gold WE of the sensor chip and incubated for 12 h at 4 °C in a humidity chamber. Then, the sensor was rinsed thoroughly with deionized water and dried under a gentle nitrogen stream. 25 μL of a 4 μM fluorescein/amine-modified aptamer solution (5’-FAM-ACG ACA TTC CGT TGA TCT CTC CCT TTT GGG TTG GTG TCG T/3AmMO/-3′) was drop-casted on the working electrode surface and left for 2.5 h at room temperature. Then, the electrode was washed thoroughly with deionized water and air-dried.

**(c):** 25 μL of a 20 μM fluorescein/amine-modified aptamer solution (5’-FAM-ACG ACA TTC CGT TGA TCT CTC CCT TTT GGG TTG GTG TCG T/3AmMO/-3′) was drop-casted on the working electrode surface and left for 2.5 h at room temperature. Then, the electrode was washed thoroughly with deionized water and air-dried.

**Circular dicroism (CD) experiments**

The experimental procedure involved recording the spectrum of the aptamer, the spectra of OTC at different concentrations and the spectra of the aptamer after addition of OTC at the same concentrations; in the last case, after addition of each OTC in the aptamer solution, a 5 min incubation period was observed. The differential spectra are obtained by subtracting the spectra of the OTC solutions from the spectra of the aptamer-OTC mixed solutions.

## **Fluorescence and fluorescence polarization titrations**

For the fluorescence and fluorescence polarization titrations, the samples were placed in 96-well microplates. Excitation was at 370 nm and emission at 530 nm. OTC and aptamer solutions were added in the microplates to prepare a range of solutions containing 200 nM OTC and 2-20000 nM of the aptamer.


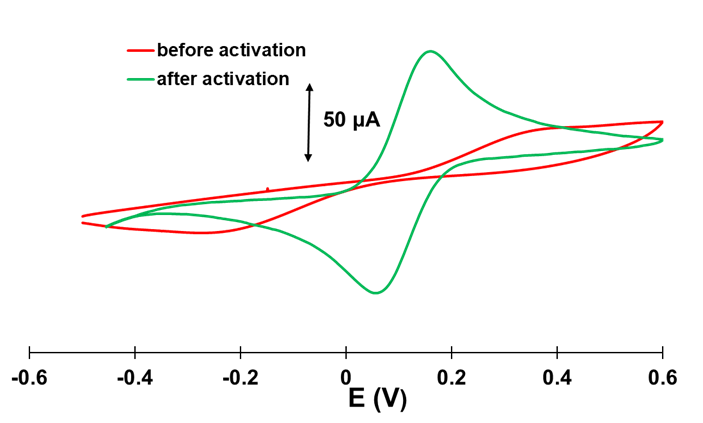


**Figure S2.** CV in a 10 mM Fe(CN)_6_^4−^/Fe(CN)_6_^3−^ solution in 0.5 M KCl before and after activation of the gold WE.


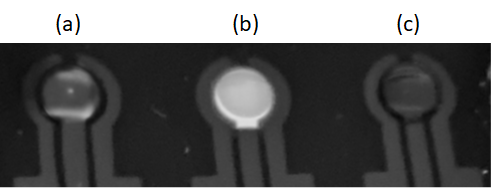


**Figure S3:** Fluorescence response of the sensor chips with the WE modified with: (a) *α*-lipoic acid and fluorescein/amine-modified aptamer, (b) *α*-lipoic acid–NHS and fluorescein/amine-modified aptamer, and (c) fluorescein/amine-modified aptamer


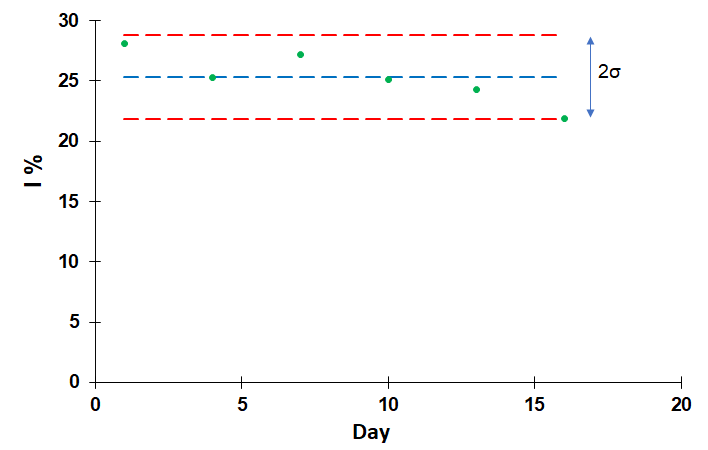


**Figure S4** Control chart illustrating the signal of a 50 ng mL^-1^ OTC standard analyzed within a fortnight on separate aptasensors prepared on day 1. The blue dotted line represents the average of the 6 measurements and the red dotted lines represent the standard deviation of the methodology.


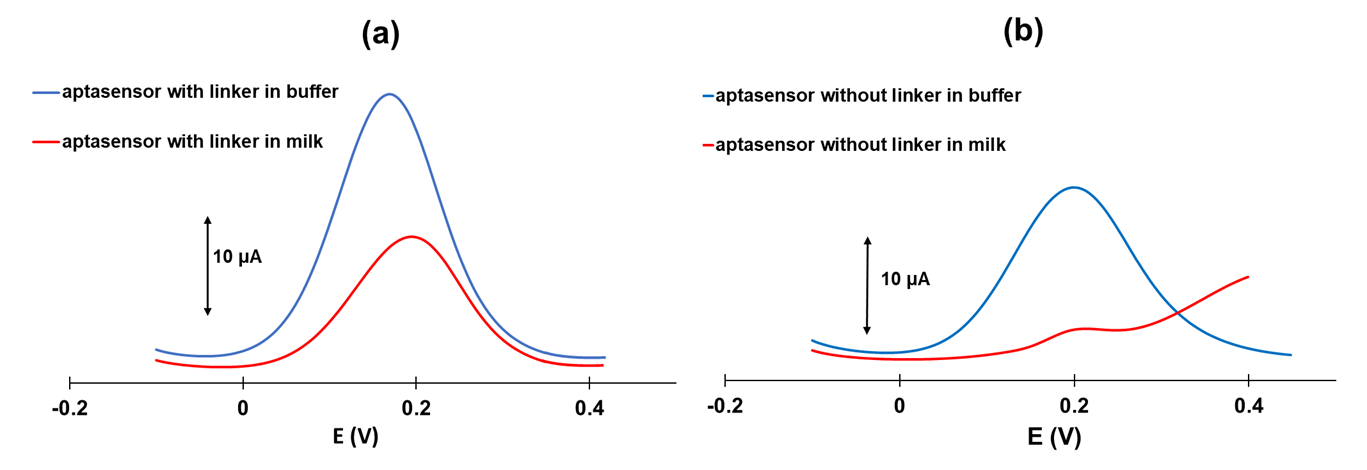


**Figure S5**. Comparison between the anodic DPVs at aptasensors in buffer and in milk (1g milk/50 mL buffer): (a) with linker using amine-modified aptamer with the protocol developed in this work, (b) without linker using thiol-modified aptamer with the protocol developed previously [54].


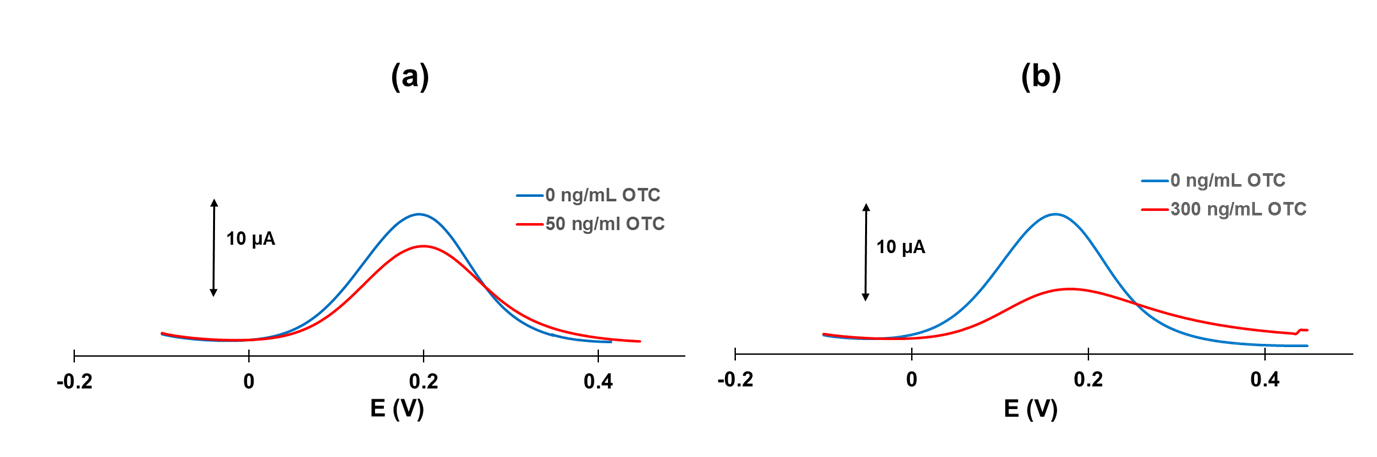


**Figure S6.** Anodic DPVs at aptasensors in milk (1g milk/50 mL buffer) before spiking with OTC and after spiking with : (a) 50 ng mL^-1^ OTC, (b) 300 ng mL^-1^ OTC.
